# Impact of Hyperuricemia on Clinical Outcomes in Sepsis Patients: A Retrospective Cohort Study

**DOI:** 10.3390/jcm13216548

**Published:** 2024-10-31

**Authors:** Abdulmajeed M. Alshehri, Mohammed Alrashed, Mohammad Shawaqfeh, Fahad Almutairi, Abdulsalam Alanazi, Mohammed Alfaifi, Nataleen A. Albekairy, Abdulrahman Alshaya, Majed S. Al Yami, Omar A. Almohammed, Shmeylan Al Harbi

**Affiliations:** 1 College of Pharmacy, King Saud bin Abdulaziz University for Health Sciences, Riyadh 11481, Saudi Arabiaamzalanazi@gmail.com (A.A.); 421110387@ksau-hs.edu.sa (M.A.);; 2 King Abdulaziz Medical City, National Guard Health Affairs, Riyadh 14611, Saudi Arabia; 3 King Abdullah International Medical Research Center, Riyadh 11481, Saudi Arabia; 4 College of Medicine, King Saud bin Abdulaziz University for Health Sciences, Riyadh 14611, Saudi Arabia; 5 Department of Clinical Pharmacy, College of Pharmacy, King Saud University, Riyadh 11451, Saudi Arabia; 6 Pharmacoeconomics Research Unit, College of Pharmacy, King Saud University, Riyadh 11451, Saudi Arabia

**Keywords:** hyperuricemia, sepsis, septic shock, mortality

## Abstract

**Background:** Hyperuricemia has been linked to various adverse clinical outcomes. Data on the clinical outcomes and the relationship between hyperuricemia and sepsis remain limited. The aim of this study was to evaluate the impact of hyperuricemia on clinical outcomes in sepsis patients and to identify whether it can predict the mortality in this patient population. **Methods:** This was a retrospective cohort study of adult patients with sepsis admitted to the intensive care unit (ICU) from 1 January 2021 to 31 December 2023. The patients were divided into hyperuricemia and non-hyperuricemia groups. Hyperuricemia was defined as a serum uric acid level > 416.4 µmol/L (7.0 mg/dL) in males or >357.0 µmol/L (6.0 mg/dL) in females based on the first serum uric acid reading within 24 h of ICU admission. The primary outcome of this study was ICU mortality. Secondary outcomes included in-hospital mortality, progression to septic shock, and ICU and hospital lengths of stay (LOSs). **Results:** A total of 599 patients were included in the study. Among these, 303 were in hyperuricemia group, while 296 were in the non-hyperuricemia group. The incidence of ICU and in-hospital mortality was higher in the hyperuricemia group compared to the non-hyperuricemia group (26.7% vs. 18.9% (*p* < 0.001) and 34.7% vs. 19.3% (*p* < 0.001), respectively). After adjusting for cofounders, hyperuricemia was not a predictor of ICU mortality (OR 1.52, 95% CI 0.95–2.43, *p* = 0.083). Most secondary outcomes were similar between the groups. However, the hyperuricemia group had a higher incidence of progression to septic shock (67.3% vs. 50.7%, *p* < 0.001), and hospital LOS was significantly longer in the hyperuricemia group (384 vs. 264 h, *p* = 0.004). **Conclusions:** Our findings demonstrated that hyperuricemia in sepsis patients was associated with worse clinical outcomes such as higher ICU and hospital mortality. Moreover, there was a higher incidence of septic shock progression and longer hospital LOS. The other outcomes were not statistically significantly different. Further prospective research is warranted to confirm these findings.

## 1. Introduction

Sepsis is a life-threatening medical condition characterized by organ dysfunction and abnormal inflammatory host responses to bacterial infections [1]. Sepsis is defined as a life-threating organ dysfunction caused by an infection or suspected infection with an increase in the Sequential Organ Failure Assessment (SOFA) score ≥ 2 [2]. It is estimated to affect over 19 million people every year around the globe, causing high mortality rates and significant healthcare costs worldwide [1,3]. Sepsis remains one of the challenging medical conditions in intensive care unit (ICUs), causing more than 30% of the short-term ICU mortality in ICUs [4]. The Surviving Sepsis Campaign’s International Guidelines for the Management of Sepsis and Septic Shock 2021 recommend hospitals and institutions to implement a sepsis performance improvement program, which includes early sepsis screening, measuring patient outcomes, and identifying opportunities for improvement in screening, identifying high-risk patients, and management. The early identification of sepsis and timely appropriate management can prevent severe complications and progression to septic shock [1]. According to the Sepsis-3 criteria, septic shock is defined as persistent hypotension that necessities vasopressor initiation to maintain a mean arterial pressure (MAP) of 65 mm Hg or greater with a serum lactate level greater than 2 mmol/L after at least 30 mL/kg of crystalloid fluid resuscitation [2]. Septic shock is a severe subset of sepsis that has a higher mortality rate compared to sepsis, ranging between 30 and 50% around the globe [5]. Significant efforts are being undertaken to optimize timely interventions and identify opportunities for the early recognition of high-risk patients, which are essential to improving both short-term and long-term clinical outcomes for these patients.

Uric acid is the end-product of purine metabolism by xanthine oxidase or xanthine dehydrogenase and is mainly excreted from the body by kidneys [6,7]. Uric acid can trigger inflammatory mediators in the epithelial cells by forming crystals in the kidneys [6]. It can also cause endothelial dysfunction, increased oxidative stress, and renal fibrosis by triggering the renin–angiotensin–aldosterone system [8]. Furthermore, uric acid has a role in activating various inflammatory transcription factors and systemic cytokine production in the kidneys [9,10]. Although the mechanisms are unclear, uric acid has been found to exert antioxidant effects at low serum levels but it promotes a pro-oxidant state at higher serum levels [11,12]. In critically ill patients, it has been reported that a time-dependent increase in uric acid can be used as a biomarker for predicting mortality [13].

Hyperuricemia is defined as an elevated serum uric acid level > 416.4 µmol/L (7.0 mg/dL) in males or >357.0 µmol/L (6.0 mg/dL) in females and results from the increased production of uric acid, decreased excretion by the kidneys, or a combination of both mechanisms [6,7]. While commonly associated with gout, growing evidence suggests that hyperuricemia might play a significant role in the pathophysiology of several diseases. It has been reported that hyperuricemia is associated with severe coronary artery disease (CAD) and could be used as a predictor of the severity of coronary artery disease in non-diabetic and non-hypertensive patients [14]. Furthermore, hyperuricemia was found to be a predictor of 30-day mortality and hospitalizations in patients with chronic obstructive pulmonary disease [15].

There is conflicting evidence about the utility of hyperuricemia as a biomarker and predictor of outcomes in critically ill patients. Some studies have linked elevated uric acid levels to a poor prognosis and worse outcomes such as increased incidences of acute injury and 90-day mortality [13,16,17,18]. Others reported that elevated uric acid is not a predictor of mortality but could be a predictor of the need for mechanical ventilation [19]. Furthermore, there is a gap in the literature and limited understanding to explain the mechanisms of how elevated uric acid levels impact the clinical outcomes in patients with sepsis and how hyperuricemia interacts with other comorbidities in sepsis patients. That said, the aim of this study was to evaluate the impact of hyperuricemia on clinical outcomes in sepsis patients and to identify whether it can predict the mortality in this patient population.

## 2. Materials and Methods

### 2.1. Patients and Study Design

This was a retrospective cohort study of adult patients with sepsis admitted to the ICU at the National Guard Health Affairs (NGHA) hospital in Riyadh, Saudi Arabia, from 1 January 2021 to 31 December 2023. The study was approved by the King Abdullah International Medical Research Center (KAIMRC) in March 2024 under reference number of NRC23R/779/12. System-generated reports using ICD-9 (038, 995.91, and 995.92) or ICD 10 (A41, R65.20, and R65.21) were used to extract reports for patients with sepsis. All identified patients were assessed to determine if they met the inclusion criteria. Patients were included if they were at least 18 years of age and met the Sepsis-3 definition of sepsis (having an infection or suspected infection with an increase in Sequential Organ Failure Assessment (SOFA) score ≥ 2) [2]. Patients were divided into hyperuricemia and non-hyperuricemia groups. Hyperuricemia was defined as a serum uric acid level > 416.4 µmol/L (7.0 mg/dL) in males or >357.0 µmol/L (6.0 mg/dL) in females based on the first serum uric acid reading within 24 h of ICU admission [16]. The baseline uric acid level was defined as the serum uric acid level collected from patients within 24 h of ICU admission. The laboratory employed a quantitative approach for reporting serum uric acid levels in the electronic health system, where results exceeding the normal range are highlighted in red to indicate abnormal levels. Patients were excluded if they were <18 years old, had no uric acid data within 24 h of admission, had a history of gout, or were on allopurinol or on hemodialysis.

### 2.2. Study Variables, Data Collection, and Outcomes

The primary outcome of this study was ICU mortality, while the secondary outcomes were in-hospital mortality; hospital and ICU lengths of stay (LOSs); progression to septic shock (defined as vasopressor initiation to maintain a mean arterial pressure (MAP) of 65 mm Hg or greater with a serum lactate level greater than 2 mmol/L after at least 30 mL/kg of crystalloid fluid resuscitation); development of acute respiratory distress syndrome (ARDS) according to the Berlin definition of ARDS; 30-day ventilation-free days (VFDs) (defined as number of days the patient is alive and does not require invasive mechanical ventilation (MV) in the first 30 days of initiating MV); and 30-day vasopressor-free days (defined as defined as number of days the patient is alive and does not require invasive vasopressors (norepinephrine, epinephrine, vasopressin, dopamine, or phenylephrine) in the first 30 days of initiating a vasopressor) [20]. Demographic and clinical data, including age, gender, body mass index, comorbidities (chronic obstructive pulmonary disease, asthma, cancer, solid organ transplant or stem cell transplant, diabetes mellitus, liver disease, and chronic kidney disease), vital signs, SOFA score, Glascow coma score (GCS), source of infection (respiratory, urinary, abdominal, skin and soft tissue, bacteremia, or other), and laboratory parameters (uric acid (µmol/L), lactate (mmol/L), platelets (×10^9^/L), serum creatinine (μmol/L), blood urea nitrogen (mmole/L), weight blood cells (cells/µL), and procalcitonin (µg/L)), were collected. The outcomes of the study were manually extracted from the electronic health system by two authors (FA and AA) and confirmed by a third author (MAF).

### 2.3. Data Analysis

Continuous variables are presented as medians (and interquartile ranges) and were analyzed using the Mann–Whitney U test while categorical data are presented as frequencies and percentages and were analyzed using Pearson’s chi-square test or Fisher’s exact test. To examine the impact of hyperuricemia on the primary outcome and to adjust for the effects of potentially confounding variables, a multivariate logistic regression was performed using variables with a *p* value of less than 0.2 in the univariate analysis. These variables included hyperuricemia, diabetes mellitus, cancer, coronary artery disease (CAD), body mass index (BMI), gender, SOFA score, MAP, and GCS. All variables with a *p* value < 0.05 were considered to have a significant impact on the outcomes. All data were analyzed using Stata/SE statistical software Version 15.1 (StataCorp LLC, College Station, TX, USA).

## 3. Results

### 3.1. Characteristics of Study Subjects

**Figure 1:**
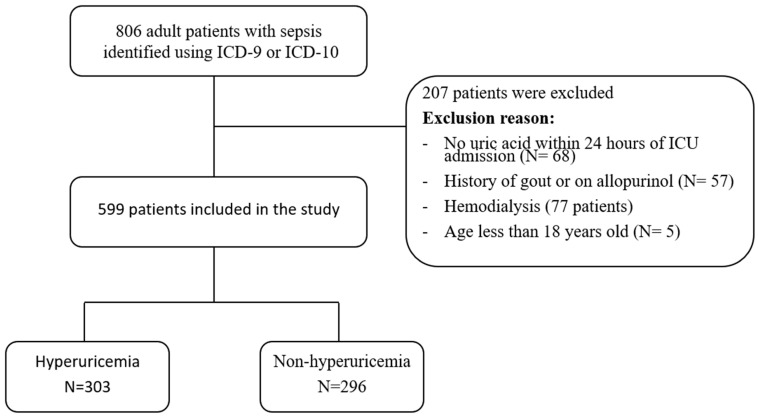
Flowchart of the included study patients.

A total of 803 adult patients with sepsis admitted to the ICU at NGHA hospital in Riyadh were identified. All identified patients were assessed based on the inclusion criteria. A total of 207 patients were excluded. Of those, 68 patients had no uric acid data within 24 h of ICU admission, 57 patients had history of gout or were on allopurinol, 77 patients were on hemodialysis, and 5 patients were younger than 18 years. A total of 599 patients were included in the study. Among these, 303 were in hyperuricemia group, while 296 were in the non-hyperuricemia group (Figure 1). A summary of the demographic and clinical characteristics is presented in Table 1. The study population primarily consisted of elderly individuals, with a median age of 71 years in both groups. The BMI of the hyperuricemia group was significantly higher (27.4 vs. 25.6, *p* = 0.001). The clinical severity of illness was higher in the hyperuricemia group, as reflected by a significantly higher median SOFA score (7 vs. 6, *p* < 0.001). The other baseline characteristics were comparable between both groups. Comorbidities in the study population were similar between both groups. The most common comorbidity was chronic kidney disease, which was present at similar rates between the groups (42.2% vs. 37.5%, *p* = 0.27), followed by diabetes mellitus (29.7% vs. 28.4%, *p* = 0.789). The sources of infection were similarly distributed between the groups, with respiratory infections being the most common source (33.7% vs. 36.8%, *p* = 0.469), followed by urinary tract infections (29% vs. 25%, *p* = 0.307). The median laboratory findings were similar between the groups except for uric acid, serum creatinine, and blood urea nitrogen which were significantly higher in the hyperuricemia group (527 vs. 258.5 μmol/L (*p* < 0.001), 189 vs. 121 μmol/L (*p* < 0.001), and 17.6 vs. 8.15 mmol/L, respectively) (Table 1).

### 3.2. Main Results

The incidence of ICU mortality was higher in the hyperuricemia group compared to the non-hyperuricemia group (26.7% vs. 18.9%, *p* < 0.001) (Table 2). After adjusting for cofounders in the multivariate logistic regression analysis, hyperuricemia was associated with a higher odds of ICU mortality, though this was not statistically significant (OR 1.52, 95% CI 0.95–2.43, *p* = 0.083). Of the variables that were included in the logistic regression analysis, the SOFA score was associated with a higher odds of ICU mortality (OR 1.24, 95% CI 1.15–1.34, *p* < 0.001). Other significant predictors of ICU mortality included cancer (OR 2.36, 95% CI 1.27–4.39, *p* = 0.007) and diabetes (OR 1.75, 95% CI 1.08–2.83, *p* = 0.023). BMI, gender, GCS, coronary artery disease, and MAP were not found to be predictors of ICU mortality (Table 3).

The incidence of in-hospital mortality was also higher in the hyperuricemia group compared to the non-hyperuricemia group (34.7% vs. 19.3%, *p* < 0.001). Similarly, the hyperuricemia group had a higher incidence of progression to septic shock (67.3% vs. 50.7%, *p* < 0.001). No significant difference was observed in the development of ARDS between the two groups (66.0% vs. 61.5%, *p* = 0.287). The median 30-day ventilation-free days was numerically lower in the hyperuricemia group compared to the non-hyperuricemia group but it did not reach statistical significance (10.5 days (IQR 1.0–25.0) vs. 18.0 days (IQR 6.0–26.0), *p* = 0.074). The median 30-day vasopressor-free days was similar between the groups (12.0 days (IQR 4.0–30.0) vs. 10.0 days (IQR 4.0–23.0), *p* = 0.15). ICU LOS was longer in the hyperuricemia group but it did not reach statistical significance (216 h (IQR 96–506) vs. 168 h (IQR 96–396), *p* = 0.152). However, hospital LOS was significantly longer in the hyperuricemia group compared to the non-hyperuricemia group (384 h (IQR 192–828) vs. 264 h (IQR 144–589), *p* = 0.004).

## 4. Discussion

The primary aim of this study was to evaluate the impact of hyperuricemia as an independent risk factor in ICU mortality in patients with sepsis. The current results suggest that hyperuricemia is associated with higher ICU mortality (*p*-value 0.029) and higher hospital mortality (*p*-value < 0.001). This study’s results demonstrate that serum uric acid levels could be potentially used as a marker as well as a predictor of the prognosis of septic patients in intensive care units.

Uric acid at physiological levels is known to have an antioxidant effect that can reduce the levels of some free radicals in the systemic circulation. However, in hyperuricemia, it will act as pro-inflammatory agent with pro-oxidant effects, thus increasing the oxidative stress. This situation ultimately leads to a cascade of events that may aid in destroying biomolecules like proteins, lipids, and nucleic acids that are involved in a variety of physiological processes [16]. The mechanisms of uric acid elevation during sepsis were not clearly elucidated in this study and this may be an unknown confounder that could be addressed with a larger sample size.

Several studies reported significant correlations between serum uric acid levels and different clinical outcomes. There was an increased hospital mortality associated with elevated levels of uric acid [21]. Similarly, increased all-cause and cardiovascular mortality rates in elderly patients with hyperuricemia were reported [22]. One study found that mortality among patients with elevated uric acid levels > 7.5 mg/dL was as high as 80% when compared to patients with uric acid levels less than 7.5 mg/dL [23]. A large study in Japan reported that all-cause and 90-day mortality and cardiovascular death in men and women were significantly higher in patients with elevated uric acid levels [24]. This conceptual study suggested a similar association between hyperuricemia and the prognosis of the disease in septic patients. However, the retrospective nature of this study as well as the moderate sample size of this single-center study hinder generalization.

Nevertheless, researchers have also evaluated hyperuricemia as a prognostic risk factor for various clinical outcomes in other disease states. The short-term mortality for myocardial infarction was significantly higher in patients with elevated serum uric acid levels [25]. A similar prognostic power for hyperuricemia was evident in cardiovascular diseases [26]. Furthermore, the mortality rate in coronary artery disease was evidently higher in patients with high uric acid serum levels [27]. Finally, one study also reported an association between hyperuricemia and 90-day mortality in acute kidney injury and diabetes patients [28].

The complex association between uric acid as antioxidant at physiological levels and as a pro-oxidant, pro-inflammatory marker when elevated was not thoroughly investigated in the septic patients with hyperuricemia. Elevated uric acid levels have been reported as a prognostic biomarker for septicemia severity in patients with hyperuricemia [13]. Elevated uric acid levels were also associated with increased 90-day and all-cause mortality rates in critically ill patients with sepsis [16]. The risk of acute kidney injury in septic patients with elevated uric acid levels was reported in several studies [18,29]. Our study results confirm an association between mortality and hyperuricemia. So, uric acid may be a potential early marker for the prognosis of septic patients.

The length of stay is an important outcome for septic patients that indicates the severity and prognosis. Patients with elevated serum uric acid levels had significantly longer lengths of hospital stay [18]. Interestingly, some studies also reported a higher Apache II score in hyperuricemia ICU patients [13,18]. Our study also reported a significantly longer length of stay in the hyperuricemia group.

However, there are a few studies that reported no association between hyperuricemia and clinical outcomes in patients with sepsis [19,30,31]. These studies only examined baseline uric acid levels in the ICU, and this might have affected their results.

Two prospective studies suggested the utilization of uric acid levels as an early marker in predicting the severity of the disease in critically ill patients with sepsis. However, these studies were limited by the small sample size of included patients with variable underlying disorders that made the risk stratification analysis very difficult [32,33].

This was retrospective single-center study that reported real-world data, and this study design is not immune from some bias; however, the reasonably convenient sample size with similar baseline characteristics minimized this limitation. There might be some unmeasured confounders such as metabolic diseases or unknown pre-admission medications that might have affected the uric acid levels. Future multi-center prospective studies may be warranted. These studies should include a larger number of patients from multiple centers with similar baseline characteristics and a longer duration of follow-up. The study can also explore the potential benefits of hyperuricemia treatment in septic patients.

## 5. Conclusions

Our findings demonstrate that hyperuricemia in sepsis patients is associated with worse clinical outcomes including ICU and hospital mortality, the development of septic shock, and prolonged hospital LOSs. However, after adjusting for cofounders in the regression analysis, hyperuricemia was not a predictor for ICU mortality. Moreover, a higher incidence of septic shock progression and a longer hospital LOS was observed in the hyperuricemia group. The other outcomes were not statistically significantly different. Further prospective research is warranted to confirm these findings on a larger scale. Our findings suggest that elevated uric acid levels may serve as a prognostic indicator in sepsis patients. Further prospective research is warranted to confirm these findings.

## Figures and Tables

**Table 1:** Baseline characteristics.

Characteristic	Hyperuricemia (n = 303)	Non-Hyperuricemia (n = 296)	*p*-Value
Age (years) *	71 (60–80.5)	71 (58.8–81)	0.894
BMI *	27.4 (22.5–33.0)	25.6 (20.8–29.8)	0.001
Male, n (%)	156 (51.5)	144 (48.6)	0.54
SOFA Score *	7 (5–10)	6 (4–8)	<0.001
GCS *	14 (10–15)	14 (10–15)	0.363
MAP *	72 (60–87)	72 (62–89)	0.844
Comorbidities, n (%)			
COPD	14 (4.6)	6 (2.0)	0.124
Asthma	27 (8.9)	17 (5.7)	0.184
Cancer	34 (11.2)	34 (11.5)	1.0
Transplant	12 (4.0)	13 (4.4)	0.952
Diabetes Mellitus	90 (29.7)	84 (28.4)	0.789
Liver Disease	27 (8.9)	32 (10.8)	0.52
Coronary Artery Disease	9 (2.9)	9 (3.04)	1.0
CKD	128 (42.2)	111 (37.5)	0.27
Source of Infection, n (%)			
Respiratory	102 (33.7)	109 (36.8)	0.469
Urinary	88 (29.0)	74 (25)	0.307
Abdominal	20 (6.6)	10 (3.4)	0.105
Skin and Soft Tissue	19 (6.3)	14 (4.7)	0.517
Bacteremia	62 (20.5)	75 (25.3)	0.186
Other	12 (4.0)	14 (4.7)	0.794
Laboratory Results at Admission *			
Uric Acid (μmol/L)	527 (432–640)	258.5 (198.5–315.3)	<0.001
Lactate (mmol/L)	2.38 (1.49–4.2)	2.12 (1.5–3.8)	0.204
Creatinine (μmol/L)	189 (122.5–333)	121 (70–292)	<0.001
CrCl (mL/min)	27 (13.5–46.5)	44 (16–78)	<0.001
BUN (mmol/L)	17.6 (11.3–27.3)	8.15 (5.3–11.6)	<0.001
WBC (cells/µL)	11.7 (7.4–17.8)	10.5 (6.7–15.3)	0.086
Platelet (×10^9^/L)	248 (157.5–359)	234 (139.5–335.8)	0.391
Procalcitonin (µg/L)	0.75 (0.25–3.86)	0.59 (0.25–4.88)	0.487

* Reported as median (interquartile range). Abbreviations: BMI, body mass index; SOFA, Sequential Organ Failure Assessment; GCS, Glasgow Coma Scale; MAP, mean arterial pressure; COPD, chronic obstructive pulmonary disease; CKD, chronic kidney disease; CrCl, creatinine clearance; BUN, blood urea nitrogen; WBC, white blood cell count. Note: Chi-square test and Mann–Whitney U test were used to compare the differences of categorical and continuous variables, respectively.

**Table 2:** Primary and secondary outcomes.

Outcome	Hyperuricemia (n = 303)	Non-Hyperuricemia (n = 296)	*p*-Value
Primary Outcome			
ICU mortality, n (%)	81 (26.7)	56 (18.9)	0.029
Secondary Outcomes			
Development of septic shock, n (%)	204 (67.3)	150 (50.7)	<0.001
Development of ARDS, n (%)	200 (66.0)	182 (61.5)	0.287
Hospital mortality, n (%)	105 (34.7)	57 (19.3)	<0.001
30-day ventilation free days *	10.5 (1.0–25.0)	18.0 (6.0–26.0)	0.074
30-day vasopressor free days *	12.0 (4.0–30.0)	10.0 (4.0–23.0)	0.15
ICU LOS (hours) *	216.0 (96.0–506.0)	168.0 (96.0–396.0)	0.152
Hospital LOS (hours) *	384.0 (192.0–828.0)	264.0 (144.0–589.0)	0.004

* Reported as median (interquartile range). Abbreviations: ARDS, acute respiratory distress syndrome; ICU, intensive care unit; LOS, length of stay. Note: Chi-square test and Mann–Whitney U test were used to compare the differences of categorical and continuous variables, respectively.

**Table 3:** Multivariate logistic regression analysis results for risk factors for ICU mortality.

Variable	Odds Ratio	95% Confidence Interval	*p* Value
Hyperuricemia	1.52	0.95 to 2.43	0.083
BMI	1.01	0.99 to 1.03	0.202
Gender	1.54	0.97 to 2.46	0.068
SOFA Score	1.24	1.15 to 1.34	<0.001
GCS	1.03	0.96 to 1.11	0.366
Cancer	2.36	1.27 to 4.39	0.007
Diabetes	1.75	1.08 to 2.83	0.023
CAD	1.56	0.5 to 4.93	0.446
MAP	1	0.99 to 1.01	0.532

Abbreviations: BMI, body mass index; SOFA, Sequential Organ Failure Assessment; GCS, Glasgow Coma Scale; CAD, coronary artery disease; MAP, mean arterial pressure.

## Data Availability

The data supporting the findings of this study will be made available by the corresponding author upon request.
